# The American Academy of Dental Science

**Published:** 1896-06

**Authors:** 


### The American Academy of Dental Science.

This society holds its regular monthly meeting about the first of each month, and sends out its programme for each meeting not only to its members but to its corresponding and honorary members as well. It always has some interesting subject for consideration, a paper on same being read, and some of the members prepared for its discussion. Usually a dinner is given just before beginning the regular work of the meeting. This is one of the model city dental societies of the country in which its members derive great benefit.

Several other cities of our country have good local societies; probably none better than this. Why should not every large city of our country have equally as good and profitable city society as Boston? Let those answer who can.

